# A Novel Monopulse Angle Estimation Method for Wideband LFM Radars

**DOI:** 10.3390/s16060817

**Published:** 2016-06-03

**Authors:** Yi-Xiong Zhang, Qi-Fan Liu, Ru-Jia Hong, Ping-Ping Pan, Zhen-Miao Deng

**Affiliations:** Department of Communication Engineering, Xiamen University, Xiamen 361005, China; 15060582203@163.com (Q.-F.L.); fandsln@163.com (R.-J.H.); pingping_pan1@163.com (P.-P.P.); dzm_ddb@xmu.edu.cn (Z.-M.D.)

**Keywords:** wideband LFM signals, monopulse angle estimation, amplitude comparison, cross-correlation, frequency estimation

## Abstract

Traditional monopulse angle estimations are mainly based on phase comparison and amplitude comparison methods, which are commonly adopted in narrowband radars. In modern radar systems, wideband radars are becoming more and more important, while the angle estimation for wideband signals is little studied in previous works. As noise in wideband radars has larger bandwidth than narrowband radars, the challenge lies in the accumulation of energy from the high resolution range profile (HRRP) of monopulse. In wideband radars, linear frequency modulated (LFM) signals are frequently utilized. In this paper, we investigate the monopulse angle estimation problem for wideband LFM signals. To accumulate the energy of the received echo signals from different scatterers of a target, we propose utilizing a cross-correlation operation, which can achieve a good performance in low signal-to-noise ratio (SNR) conditions. In the proposed algorithm, the problem of angle estimation is converted to estimating the frequency of the cross-correlation function (CCF). Experimental results demonstrate the similar performance of the proposed algorithm compared with the traditional amplitude comparison method. It means that the proposed method for angle estimation can be adopted. When adopting the proposed method, future radars may only need wideband signals for both tracking and imaging, which can greatly increase the data rate and strengthen the capability of anti-jamming. More importantly, the estimated angle will not become ambiguous under an arbitrary angle, which can significantly extend the estimated angle range in wideband radars.

## 1. Introduction

Angle estimation is about estimating the direction of arrivals of the received radar signals. Traditional monopulse angle estimation methods can be classified into two types: amplitude comparison methods [1,2,3,4,5] and phase comparison methods [3,4,5,6,7,8,9,10,11]. In amplitude comparison methods, the ratio of the difference channel output to the sum channel output (commonly called the delta-over-sum (DOS) ratio) is first calculated. The angle of arrival (AOA) is then estimated according to the known relationship between the DOS ratio and a target angle. However, under noisy circumstances, the DOS ratio may be biased and the angular accuracy of amplitude comparison could be relatively low.

In monopulse phase comparison methods, angle estimation is implemented by measuring the phase difference between the received signals of two antennas or two identical antenna elements. The target angle can then be calculated according to the relationship between the phase difference and the antenna spacing. Although the accuracy of phase comparison angle estimation is high, it is sensitive to noise, especially in low signal-to-noise ratio (SNR) conditions. Moreover, when the phase difference is larger than 2π, the estimated angle will be ambiguous.

The two angle estimation methods above are commonly adopted in narrowband radars. Since range resolution is normally inversely proportional to the bandwidth of the transmitted pulses, wideband radars can be capable of forming the high resolution range profile (HRRP) of a target from the radar returns [12,13,14]. Due to the high range resolution of wideband signals, the target may occupy multiple range cells. If the traditional monopulse angle estimations are used for wideband signals, only the dominant scatterer of the target can be used to estimate the direction of arrivals. This means that the energy scattering from the target cannot be accumulated and may lead to the loss of SNR. Although the HRRP may be beneficial to estimating the target angle, angle estimation used for wideband signals is little investigated in previous works.

Wideband radars transmit wideband waveforms, such as direct short pulses, digital phase coding signals, discrete frequency coding signals, and linear frequency modulated (LFM) pulses. This paper focuses on the angle estimation for wideband LFM signals and a novel monopulse angle estimation algorithm is proposed. By performing cross-correlation operation to the signals received by different parts of an antenna, the energy of the received echo signals from different scatterers of a target can be accumulated, and the AOA can be estimated from the cross-correlation function (CCF). In the proposed algorithm, the original angle estimation problem is converted to estimating the frequency of the CCF. More importantly, since the impact of noise on the frequency of a sinusoid is much less than the phase impact, the proposed algorithm can work well in low SNR conditions.

The rest of this paper is organized as follows. The conventional monopulse amplitude comparison angle estimation is first presented in Section 2. In Section 3, the proposed monopulse angle estimation method based on cross-correlation operation is introduced. The implementation of the proposed method is discussed in Section 4. The SNR analysis is given in Section 5, and the simulation results are shown in Section 6. Finally, we conclude in Section 7.

## 2. Conventional Monopulse Amplitude Comparison Angle Estimation

**Figure 1:**
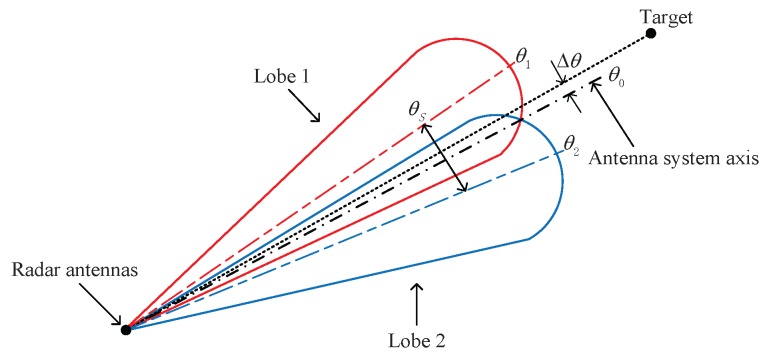
Partial antenna patterns for amplitude comparison monopulse system.

Before we introduce the proposed method, we briefly discuss the principles of the classical monopulse amplitude comparison angle estimation in this section. As shown in Figure 1, the amplitude comparison monopulse system should have *X* equal antennas with overlapping patterns, where the two main beams are squinted at a certain angle $\theta_{s}$, and usually in such a way that they overlap at the 3 dB-point of the beams.

Two resulting patterns (*i.e.*, sum pattern and difference pattern) are obtained by adding and subtracting the signals from the *X* antennas. The received sum signal is used for target detection, range measurement, and as the phase reference for determining the sign of angle error measurement. The magnitude of the angle error Δθ is determined by the difference signal. The angle error is measured in reference to the antenna system axis $\theta_{0}$.

In the following, the computation of the error signals is presented. Without loss of generality, the elevation error signal is considered. The signals received from *X* antennas are $S=\left[S_{1},S_{2},\cdots ,S_{X}\right]$, where the signal *S* is an X×1 dimensional matrix. Suppose that the weights for the phase shift of the sum pattern are $W_{\sum }=\left[W_{1},W_{2},\cdots ,W_{X}\right]$, where the weights $W_{\sum }$ is a 1×X dimensional matrix. Then, the output of the sum pattern is $F_{\sum }=W_{\sum }S$. The weights for the phase shift of the two overlapping patterns pointing to $\theta_{1}=\theta_{0}-\theta_{s}/2$ and $\theta_{2}=\theta_{0}+\theta_{s}/2$ are $W_{L}$ and $W_{R}$, respectively. Thus, the outputs of the two overlapping patterns are $F_{L}=W_{L}S$ and $F_{R}=W_{R}S$, respectively, and the output of the difference pattern is $F_{\Delta }=F_{L}-F_{R}$. If $F_{L}\geq F_{R}$, then both channels have the same phase $0^{\circ }$. Alternatively, if $F_{L}<F_{R}$, then the two channels are $180^{\circ }$ out of phase. Thus, the error signal output is

(1) $$\varepsilon =\frac{\left|F_{\Delta }\right|}{\left|F_{\sum }\right|}\cos\xi$$

where *ξ* is the phase angle between the sum and difference channels, and it is equal to $0^{\circ }$ or $180^{\circ }$.

In practice, however, the DOS ratio may be biased under the influence of noise. In addition, angle estimation can only take place within a narrow region near the boresight.

## 3. Monopulse Angle Estimation Based On Cross-Correlation Operation

### 3.1. Received Signal Model

Due to the high range resolution of wideband signals, the target may occupy multiple range cells. When using the amplitude comparison based on wideband signals, the boresight can only point to the dominant scatterer of the target, which may lead to the loss of SNR. Moreover, if there is no dominant scatterer in the HRRP, the amplitude comparison may not work correctly. Therefore, we propose a novel monopulse angle estimation based on cross-correlation operation to solve these problems.

**Figure 2:**
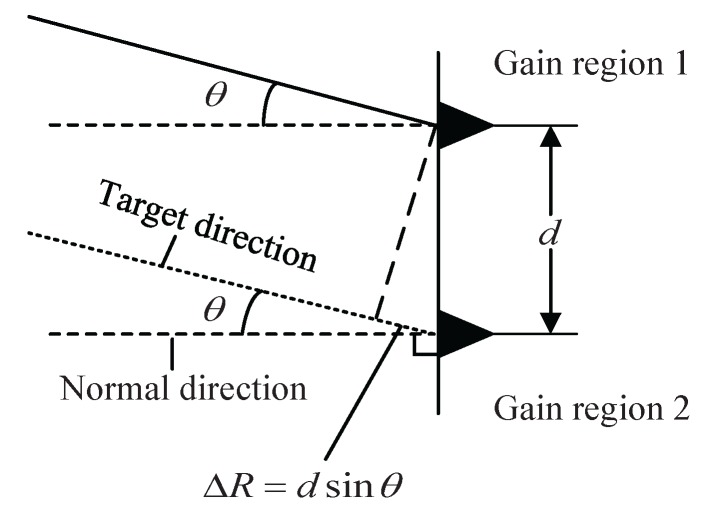
The plane model of the proposed method on planar array antenna.

In wideband radars, LFM signals are transmitted. The range resolution of the target is c/2B, where *c* is the velocity of light and *B* is the signal bandwidth. The chirp rate is k=B/T, where *T* is the LFM pulse width. Figure 2 is the plane model of the proposed method on planar array antenna. Suppose that a far-field target lies in the direction of *θ*, and the returned electromagnetic wave can be approximated as a plane wave. The normal direction is perpendicular to the array antenna plane, and the spacing between two gain regions is *d*. Since we focus on the angle estimation of phased array radars, we will use the term “gain region” instead of “antenna element” in the following.

Due to the high range resolution of wideband LFM signals, the target may occupy multiple range cells. Assume that the radar receives echo signals from a target containing *P* scatterers. The echo signals after dechirp and filtering can be expressed as

(2) $$r\left(t+t_{m}\right)=\sum_{p=1}^{P}A_{p}e^{j\left(-2\pi f_{c}T_{p}\left(t+t_{m}\right)+\pi k\left(-2tT_{p}\left(t+t_{m}\right)+T_{p}{\left(t+t_{m}\right)}^{2}\right)+\theta_{c}\right)}+w\left(t+t_{m}\right),m=1,...,M$$

where *p* is the scatterer index, $A_{p}$ denotes the signal strength of the *p*-th scatterer, and $w\left(t\right)$ is the additive white Gaussian noise, of which the mean value is 0 and the variance is $\sigma^{2}$. Since the processes of dechirp, filtering and analog to digital acquisition are completed independently for each channel, the noises of different channels are assumed to be independent in this paper. $f_{c}$ and $\theta_{c}$ denote the carrier frequency and the initial phase of the echo signals, respectively. $T_{p}\left(t+t_{m}\right)$ denotes the round trip time for the *p*-th scatterer. *t* is the fast time and $t_{m}$ is the slow time. *m* denotes the pulse number, and *M* is the total number of pulses.

Note that the proposed algorithm is a monopulse angle estimation method. For the echo signals of different scatterers, the slow time $t_{m}$ is the same, while the round trip times $T_{p}\left(t\right)$ are different. Thus, for monopulse, Equation (2) can be simplified as

(3) $$r_{i}\left(t\right)=\sum_{p=1}^{P}A_{p}e^{j\left(-2\pi f_{c}T_{ip}\left(t\right)+\pi k\left(-2tT_{ip}\left(t\right)+T_{ip}{\left(t\right)}^{2}\right)+\theta_{c}\right)}+w\left(t\right)$$

where $r_{i}\left(t\right)\left(i=1,2\right)$ denotes the target echo signal received by gain region *i*. $T_{ip}\left(t\right)$ denotes the round trip time of the signal from the gain region *i* to the *p*-th scatterer.

In this paper, we adopt a stop-and-go model [15]. Hence, in a pulse interval, $T_{ip}\left(t\right)$ can be expressed approximately as

(4) $$T_{ip}\left(t\right)=2\tau_{ip}$$

where $\tau_{ip}=R_{ip}/c$, $R_{ip}$ is the instantaneous distance from the gain region *i* to the *p*-th scatterer.

### 3.2. Cross-Correlation of the Received Signals

Applying cross-correlation operation to the echo signals received separately by gain regions 1 and 2, we can get the function $CR\left(t\right)$ as follows:

(5) $$\begin{array}{cc} CR\left(t\right) & =r_{1}\left(t\right)r_{2}^{*}\left(t\right)\\ & =\sum_{p=1}^{P}A_{p}^{2}e^{j\left(-4\pi f_{c}\left(\tau_{1p}-\tau_{2p}\right)+\pi k\left(\left(-4t\tau_{1p}+4\tau_{1p}^{2}\right)-\left(-4t\tau_{2p}+4\tau_{2p}^{2}\right)\right)\right)}\\ & \;+\sum_{p=1}^{P}\sum_{q=1}^{P}A_{p}A_{q}e^{j\left(-4\pi f_{c}\left(\tau_{1p}-\tau_{2q}\right)+\pi k\left(\left(-4t\tau_{1p}+4\tau_{1p}^{2}\right)-\left(-4t\tau_{2q}+4\tau_{2q}^{2}\right)\right)\right)}+w_{C}\left(t\right)\\ & =CR_{S}\left(t\right)+CR_{I}\left(t\right)+w_{C}\left(t\right) \end{array}$$

where superscript * denotes the conjugate operation. The first term $CR_{S}\left(t\right)$ is the signal term. For p=q, we have

(6) $$CR_{S}\left(t\right)=\sum_{p=1}^{P}A_{p}^{2}e^{j\left(-4\pi f_{c}\left(\tau_{1p}-\tau_{2p}\right)+\pi k\left(\left(-4t\tau_{1p}+4\tau_{1p}^{2}\right)-\left(-4t\tau_{2p}+4\tau_{2p}^{2}\right)\right)\right)}$$

The second term $CR_{I}\left(t\right)$ is the interference term. For p≠q, we have

(7) $$CR_{I}\left(t\right)=\sum_{p=1}^{P}\sum_{q=1}^{P}A_{p}A_{q}e^{j\left(-4\pi f_{c}\left(\tau_{1p}-\tau_{2q}\right)+\pi k\left(\left(-4t\tau_{1p}+4\tau_{1p}^{2}\right)-\left(-4t\tau_{2q}+4\tau_{2q}^{2}\right)\right)\right)}$$

and $w_{C}\left(t\right)$ is the noise term after cross-correlation.

The signal term $CR_{S}\left(t\right)$ can be re-written as

(8) $$CR_{S}\left(t\right)=\sum_{p=1}^{P}A_{p}^{2}e^{j\left(2\pi f^{\prime }t+\theta^{\prime }\right)}$$

where

(9) $$f^{\prime }=2k\left(\tau_{2p}-\tau_{1p}\right)$$

(10) $$\theta^{\prime }=4\pi f_{c}\left(\tau_{2p}-\tau_{1p}\right)+4\left(\tau_{1p}^{2}-\tau_{2p}^{2}\right)\pi k$$

We can see that the signal term $CR_{S}\left(t\right)$ is a one-order exponential function of *t* and has two exponential terms in Equation (8). The first term, which depends on $f^{\prime }$, is a linear function of the fast time *t*. The second term is independent of the fast time.

As the transmitted signals can be approximated as plane waves, the propagation time difference between the two round trip times $\tau_{2p}-\tau_{1p}$ is the same for every scatterer of the same target. In other words, $\tau_{2p}-\tau_{1p}=\Delta \tau =d\sin\theta /c$, for p=1,...,P. Thus, $f^{\prime }$ and $\theta^{\prime }$ can be simplified as

(11) $$f^{\prime }=2k\Delta \tau$$

(12) $$\theta^{\prime }=4\pi f_{c}\Delta \tau +4\left(\tau_{1p}^{2}-\tau_{2p}^{2}\right)\pi k$$

For p≠q, the interference term of $CR\left(t\right)$ can also be re-written as

(13) $$CR_{I}\left(t\right)=\sum_{p=1}^{P}\sum_{q=1}^{P}A_{p}A_{q}e^{j\left(2\pi f^{\prime \prime }t+\theta^{\prime \prime }\right)}$$

where

(14) $$f^{\prime \prime }=-2k\left(\tau_{1p}-\tau_{2q}\right)$$

(15) $$\theta^{\prime \prime }=-4\pi f_{c}\left(\tau_{1p}-\tau_{2q}\right)+4\left(\tau_{1p}^{2}-\tau_{2q}^{2}\right)\pi k$$

Generally, the radial distances are different for different scatterers. Due to the different combinations of *p* and *q*, the time delay elements $\tau_{1p}-\tau_{2q}\left(p=1,...,P\;,\;q=1,...,P\right)$ of Equation (14) will bring many frequency components in the spectra of the CCF. For p=q, the energy of all scatterers is accumulated on the main spectral line $f^{\prime }$. For p≠q, the energy is dispersed on other spectral lines $f^{\prime \prime }$, of which the amplitudes are much less than that of the main spectral line $f^{\prime }$.

The frequency of the main spectral line can be estimated by fast Fourier transform (FFT) or other frequency estimation methods. The propagation time difference Δτ of the two gain regions can be calculated according to $\Delta \tau =f^{\prime }/2k=d\sin\theta /c$. Therefore, the target angle can be estimated according to

(16) $$\theta =\arcsin\left(\frac{cf^{\prime }}{2kd}\right)$$

Applying differently to $cf^{\prime }/2kd=\sin\theta$, we can obtain

(17) $$\frac{c}{2kd\cos\theta }df^{\prime }=d\theta$$

From Equation (17), given observed values $df^{\prime }$, the estimated angular accuracy is inversely proportional to cosθ. When θ=0, the target is located in the normal direction of the antenna elements, so the estimation error dθ is minimized. When the target angle *θ* increases, the estimation error dθ also increases. In addition, the estimated angular accuracy is inversely proportional to the chirp rate k. The larger k is, the higher the estimation accuracy. For two pulses with the same pulse width and different bandwidth, the chirp rate of wideband pulse is much larger than that of narrowband pulse. Therefore, the proposed method is effective for wideband LFM signals.

**Figure 3:**
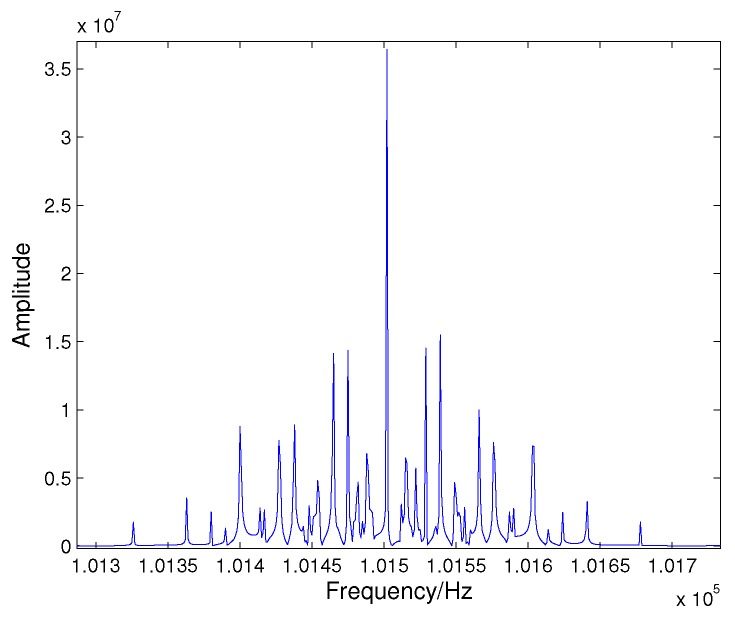
The spectra of the cross-correlation function (CCF) for a target containing 17 scatterers.

An example of the spectra of the CCF is shown in Figure 3. The echo signals are reflected by a target containing 17 scatterers. From Figure 3, for this target, the energy of the received echo signals after cross-correlation operation is concentrated. Obviously, the more concentrated the signal energy is, the higher the estimated frequency accuracy will be. Therefore, the proposed algorithm can achieve good performance in low SNR conditions.

## 4. Implementation of The Proposed Method on Phased Array Radars

From the theoretical analysis above, the proposed cross-correlation algorithm can be used to estimate a target’s elevation angle and azimuth angle.

**Figure 4:**
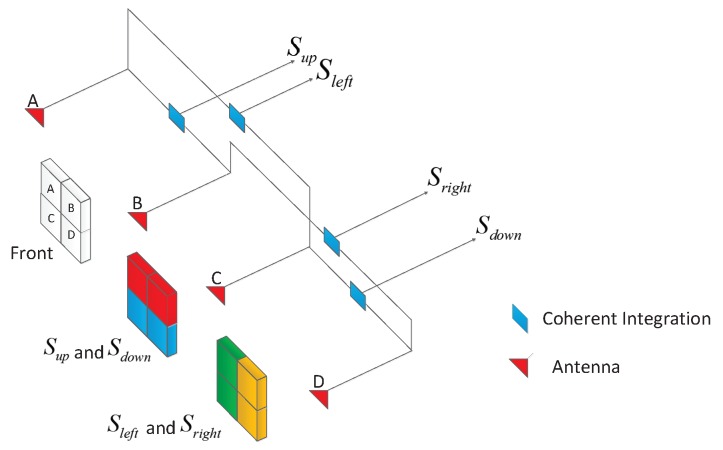
The coherent integration operation for angle estimation.

**Figure 5:**
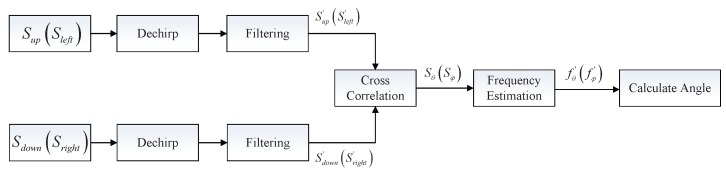
The structure of the proposed monopulse angle estimation.

First, in phased array radars, the LFM echo signals are received, respectively, by four quadrants, which can be regarded as four identical antenna elements. These four signals will be coherently integrated to form four gain region signals (*i.e.*, $S_{up}$, $S_{down}$, $S_{left}$, $S_{right}$). It should be noted that the gain region can be formed via beamforming, thus the proposed method can also be applied in radar systems using beamforming. The coherent integration operation for angle estimation is shown in Figure 4. Figure 5 shows the structure of the proposed monopulse angle estimation. Dechirp and filtering are generally applied to each signal. After dechirp and filtering, the sampling frequency of echo signals will decrease, and we can get four signals (*i.e.*, $S_{up}^{\prime }$, $S_{down}^{\prime }$, $S_{left}^{\prime }$, $S_{right}^{\prime }$).

Then, we perform cross-correlation operation as Equation (5), and two cross-correlation functions are obtained. $S_{\theta }=S_{up}^{\prime }\cdot S_{down}^{\prime *}$ is for the elevation angle and $S_{\varphi }=S_{left}^{\prime }\cdot S_{right}^{\prime *}$ is for the azimuth angle.

Finally, frequency estimation methods, for example, FFT and Newton iteration [16,17], can be utilized to estimate the peak spectrum positions of $S_{\theta }$ and $S_{\varphi }$.

Suppose that the frequencies of the peak spectrum position are $f_{\theta }^{\prime }$ and $f_{\varphi }^{\prime }$, respectively. The estimated elevation angle and azimuth angle can then be calculated as follows:

(18) $$\theta^{\prime }=\arcsin\left(\frac{f_{\theta }^{\prime }c}{2kd}\right)$$

(19) $$\varphi^{\prime }=\arcsin\left(\frac{f_{\varphi }^{\prime }c}{2kd}\right)$$

## 5. SNR Analysis for Angle Estimation

### 5.1. Threshold SNR

The SNR of wideband returned signal is defined as in [18,19,20]. The threshold SNR of angle estimation based on wideband radars is mainly affected by two factors. First, compared with narrowband radars, the noise in wideband radars has a larger bandwidth. Second, cross-correlation operation on the echo signals may result in the loss of SNR.

An echo signal $r_{i}\left(t\right)$ can be expressed as

(20) $$r_{i}\left(t\right)=s_{i}\left(t\right)+w_{i}\left(t\right)$$

where $s_{i}\left(t\right)$ is the received signal, whose amplitude is *A*. $w_{i}\left(t\right)$ is the noise, whose mean and variance are 0 and $\sigma^{2}$, respectively. The SNR of $r_{i}\left(t\right)$ is defined as $SNR_{i}=A^{2}/\sigma^{2}$.

The CCF of the two received signals is

(21) $$\begin{array}{c} r_{1}\left(t\right)r_{2}^{*}\left(t\right)=s_{1}\left(t\right)s_{2}^{*}\left(t\right)+s_{1}\left(t\right)w_{2}^{*}\left(t\right)+s_{2}^{*}\left(t\right)w_{1}\left(t\right)+w_{2}^{*}\left(t\right)w_{1}\left(t\right) \end{array}$$

The signal term is $s\left(t\right)=s_{1}\left(t\right)s_{2}^{*}\left(t\right)$, and the noise term is $w\left(t\right)=s_{1}\left(t\right)w_{2}^{*}\left(t\right)+s_{2}^{*}\left(t\right)w_{1}\left(t\right)+w_{2}^{*}\left(t\right)w_{1}\left(t\right)$. The mean and variance of w(t) are 0 and $2A^{2}\sigma^{2}+\sigma^{4}$, respectively. The SNR after cross-correlation is

(22) SNRc=A42A2σ2+σ4=SNRi2+11SNRiSNRi

**Figure 6:**
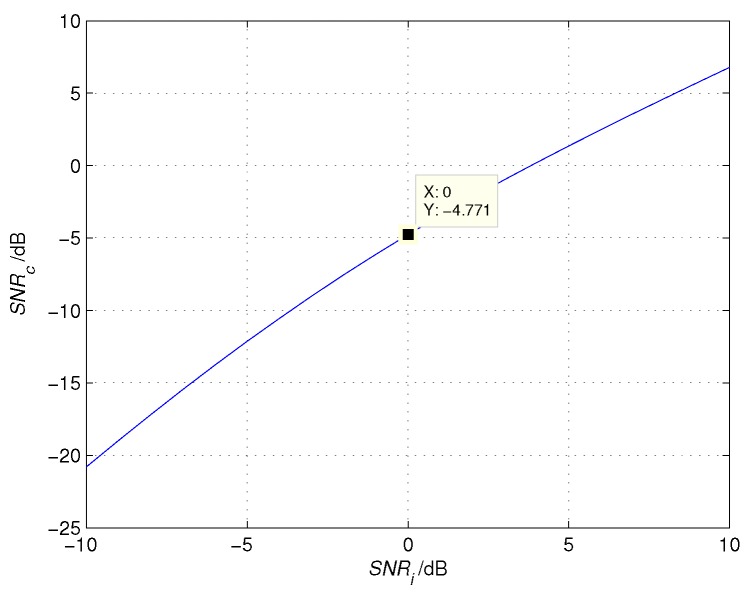
The relationship between the SNR after cross-correlation ($SNR_{c}$) and the SNR of echo signal ($SNR_{i}$).

Equation (22) shows that the SNR decreases after cross-correlation. The relationship between $SNR_{c}$ and $SNR_{i}$ is depicted in Figure 6. For example, $SNR_{c}$ is −4.7 dB when $SNR_{i}$ is 0 dB. When $SNR_{i}<0$ dB, the loss rate increases gradually with the decrease of $SNR_{i}$.

Because angle estimation is based on estimating the frequency of the highest spectral line in the spectrum, the accumulated SNR of the CCF after FFT should be more than 13.4 dB, according to the threshold SNR of spectrum estimation [21]. Suppose that the sampling number of the echo signal is *N*, and the SNR after cross-correlation is $SNR_{c}$. Then, the threshold SNR of angle estimation should satisfy

(23) $$10\log_{10}\left(N\cdot SNR_{c}\right)\geq 13.4$$

### 5.2. Root Mean Square Error

From the previous analysis, the angle estimation can be simplified as frequency estimation of a sinusoid, after the cross-correlation operation. For a sinusoidal signal, if the frequency, amplitude and phase are unknown [21], the root mean square error (RMSE) of frequency estimation is

(24) $$RMSE\left(f\right)=\sqrt{\frac{6}{4\pi^{2}SNRT_{s}^{2}N\left(N^{2}-1\right)}}$$

where SNR is the SNR of the signal, $T_{s}$ is the sampling interval, and *N* is the sampling number.

Substituting Equation (24) into Equation (17), and letting SNR be $SNR_{c}$, the RMSE of *θ* can be written as

(25) $$RMSE\left(\theta \right)=\frac{c}{2kd\cos\theta }\sqrt{\frac{6}{4\pi^{2}SNR_{c}T_{s}^{2}N\left(N^{2}-1\right)}}$$

## 6. Simulation Results

In this section, we conduct the elevation angle estimation through simulations to verify the effectiveness of the proposed approach. The estimation of azimuth angle is similar and omitted here. The detail setting of the simulation is shown as follows. The wideband radar transmits chirp signals with bandwidth B=1 GHz. The center frequency is $f_{c}=9$ GHz and the pulse duration is T=200μs. Suppose there is a far-field target containing 17 scatterers and the length of the target is about 13 m, and the target range is 10,000 m. The spacing between two gain regions is 2 m. In the process of signal processing, after dechirp and filtering, the sampling frequency of echo signals will decrease to 10 MHz. We utilize the Newton iteration to estimate the peak spectrum position. All results were conducted with 1000 Monte Carlo simulations.

**Figure 7:**
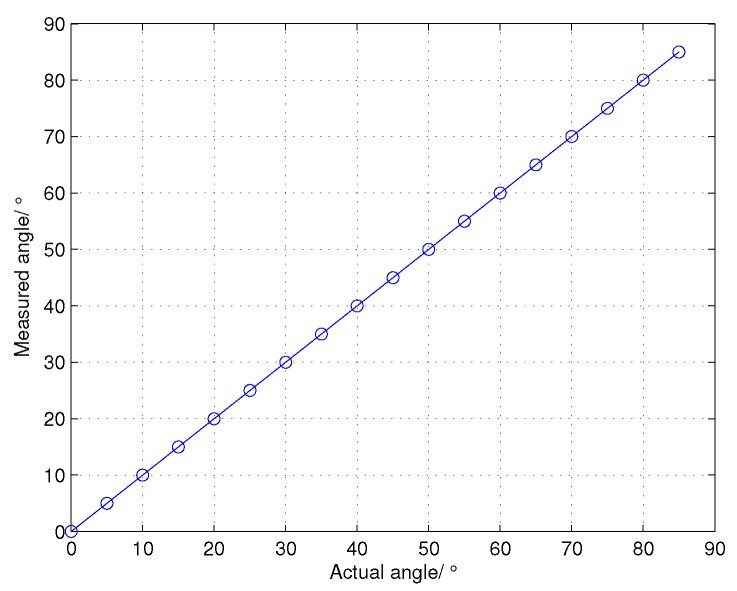
The performances of the cross-correlation angle estimation (CCAE) in different target angles.

**Figure 8:**
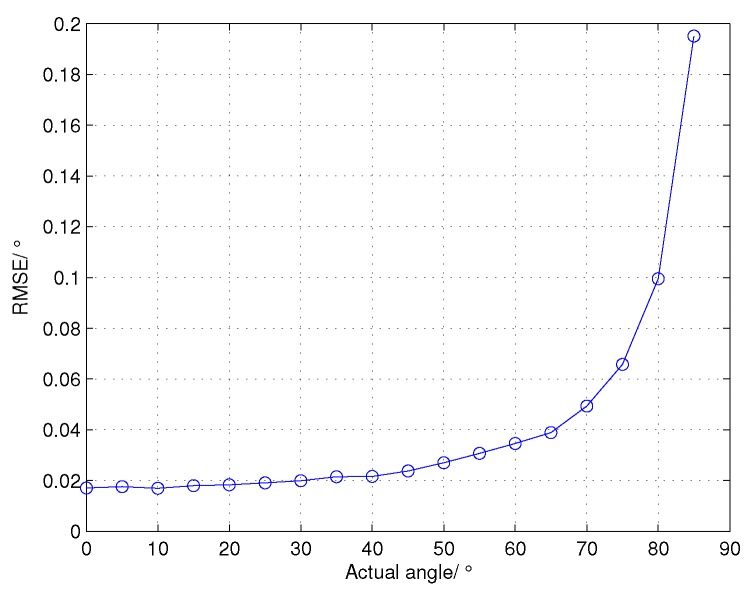
The root mean square error (RMSE) of CCAE in different target angles.

The performances of the proposed cross-correlation angle estimation (CCAE) in different target angles are shown in Figure 7. The actual elevation angle of the target is supposed to vary from $0^{\circ }$ to $85^{\circ }$ in steps of $5^{\circ }$, and the SNR of the input dechirped signal is 10 dB. We can see that there is no angle ambiguity for the CCAE. Thus, the estimated angle range in wideband radars can be significantly extended. Figure 8 shows the RMSE of CCAE in different target angles. With the increase of the target angle, the RMSE also grows gradually. This result can be explained by Equation (17) and is consistent with the theoretical analysis at the end of Section 3. Even though the RMSE grows when the target angle increases, the errors are still within the acceptable range.

**Figure 9:**
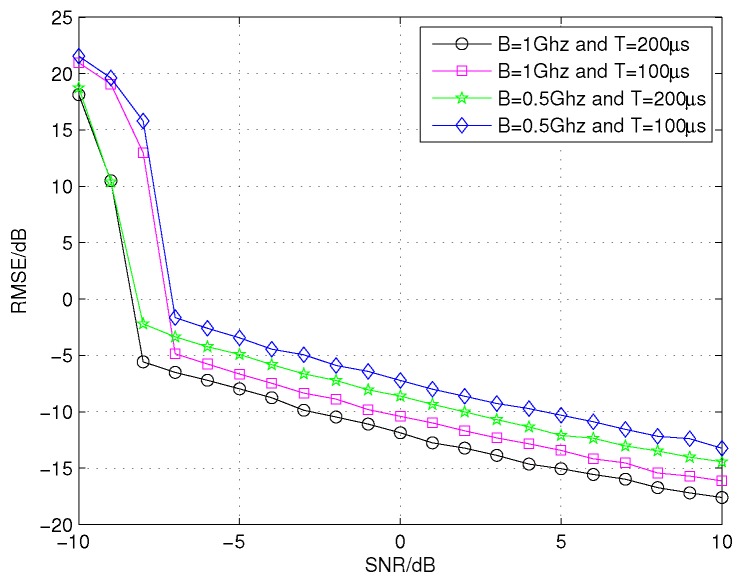
The performances of the CCAE with different values of bandwidth and pulse durations in different SNR conditions.

We now compare the performances of the proposed CCAE with different bandwidths and pulse durations in different SNR conditions. Assume that the elevation angle of the above 17 scatterers target is $0.2^{\circ }$, and the SNR varies from −10 dB to 10 dB in steps of 1 dB. As shown in Figure 9, to achieve the same value of RMSE, the SNR needed by the CCAE with B=1 GHz and T=200μs is the lowest and its performance is the best. When the SNR is the same, the difference of the RMSE between the case with B=1 GHz and T=200μs and the case with B=0.5 GHz and T=200μs is about 3 dB, which is similar to the theoretical value 3.0103 dB; the difference of the RMSE between the case with B=1 GHz and T=200μs and the case with B=1 GHz and T=100μs is about 1.5 dB, which is similar to the theoretical value 1.5051 dB. These results are consistent with the theoretical analysis and verify the effectiveness of the approximated RMSE, that is, Equation (25).

**Figure 10:**
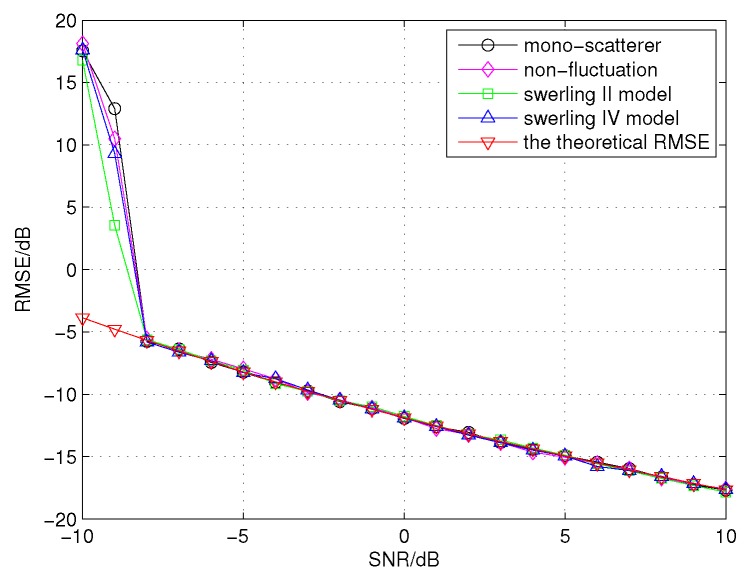
The performances of the proposed algorithm for different types of targets and the theoretical RMSE.

In the following, we evaluate the performances of the proposed algorithm for different types of targets. Four targets are considered, namely mono-scatterer target, non-fluctuation target containing 17 scatterers, Swerling II target containing 17 scatterers and Swerling IV target containing 17 scatterers. In this simulation, assume that the target elevation angle is $0.2^{\circ }$, and the SNR varies from −10 dB to 10 dB in steps of 1 dB. The total signal power of each echo signal for different types of targets is normalized to 1. From Figure 10, the threshold SNR and the RMSE of the three targets containing 17 scatterers are nearly the same. Since the CCAE is a monopulse angle estimation method, the simulation of the Swerling I target is equivalent to the Swerling II target, and the simulation of the Swerling III target is equivalent to the Swerling IV target, which are omitted here. These results indicate that the CCAE is not sensitive to the types of targets. Moreover, Figure 10 also shows that the performance of the mono-scatterer target is almost the same as those of the targets containing 17 scatterers, which means that the proposed method can accumulate the energy of the received echo signals from different scatterers of a target.

Figure 10 shows that the RMSE of the simulation result and the theoretical RMSE result are close. Furthermore, the threshold SNR of the simulation result (approximated −8 dB) and the theoretical threshold SNR calculated by Equations (22) and (23) (approximated −9.35 dB) are also close. The difference between the theoretical threshold SNR and the simulation result is only about −1.35 dB.

**Figure 11:**
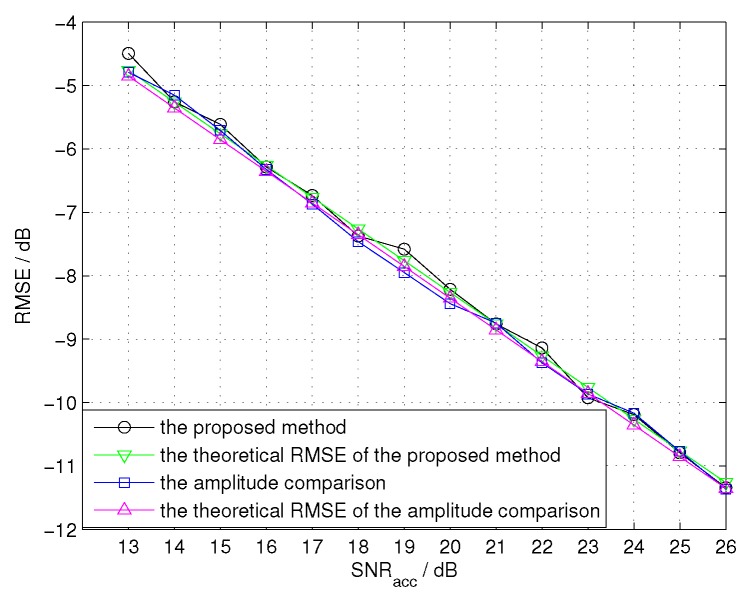
The performances of the amplitude comparison angle estimation (ACAE) and CCAE for the target containing 17 scatterers in different SNR conditions.

Finally, we compare the performances of the proposed CCAE with that of the amplitude comparison angle estimation (ACAE) in different SNR conditions. We evaluate the ACAE in narrowband condition, that is, the bandwidth is 10 MHz, the sampling frequency is 10 MHz, and other parameters remain the same. Since the parameters in narrowband conditions are different from those in wideband condition, we compare the performances of two methods in the same accumulated SNR, which is represented by $SNR_{acc}$. As 13 dB is the threshold SNR of signal detection, we take it as a starting point, and the accumulated SNR is supposed to vary from 13 dB to 26 dB in the steps of 1 dB. Then, Equation (25) should be changed as $\frac{c}{2kd\cos\theta }\sqrt{\frac{6}{4\pi^{2}SNR_{acc}T_{s}^{2}\left(N^{2}-1\right)}}$, where $SNR_{acc}=SNR_{c}\cdot N$. The theoretical RMSE of ACAE is obtained by $\frac{\theta_{\frac{1}{2}}}{K_{m}\sqrt{2SNR_{acc}}}$ [22], where $\theta_{\frac{1}{2}}$ is 3 dB of the beam width, and $K_{m}$ is the normalized monopulse slope. In this simulation, $\theta_{\frac{1}{2}}$ is equal to $1.8540^{\circ }$, and $K_{m}$ is equal to 1.1694. From Figure 11, we find that the performances of two methods are similar, which verify the feasibility of CCAE. Furthermore, according to Equation (25), the performance of the proposed method depends on three parameters including the chirp rate, the spacing between two gain regions and the sampling number. The larger the chirp rate, the larger the spacing between the two gain regions, or the higher the sampling number, the better performance the proposed method can achieve. Therefore, the performance of the proposed method will be better than the ACAE method, when the chirp rate or the spacing of two gain regions is relatively large.

Due to the high range resolution of wideband signals, the target may occupy multiple range cells. As we know, ACAE is about estimating the angle of the strongest scatterer in the main lobe of the antenna. When using ACAE for wideband signals, the angle estimation should only be based on one of the dominant scatterers of the target. This will lead to the loss of SNR. Moreover, if there is no dominant scatterer in the HRRP, the ACAE may not work correctly. Note that the angle estimation for wideband radars is investigated little in previous works. In modern radar systems, narrowband signals are usually used for angle estimation and object tracking, while wideband signals are used for imaging. Therefore, narrowband signals and wideband signals should be transmitted alternatively. When adopting the proposed CCAE, future radars may only need wideband signals for both tracking and imaging, which can greatly increase the data rate and strengthen the capability of anti-jamming.

## 7. Conclusions

In this paper, we investigate monopulse angle estimation for wideband LFM signals. Due to the high range resolution of wideband signals, the target may occupy multiple range cells. If the traditional monopulse angle estimations are used for wideband signals, only the dominant scatterer of the target can be used to estimate the direction of arrivals. This means that the energy scattering from the target cannot be accumulated and may lead to the loss of SNR. Moreover, if there is no dominant scatterer in the HRRP, the traditional monopulse angle estimations may not work correctly. To solve these problems, we propose a novel monopulse angle estimation algorithm based on cross-correlation operation. In the proposed algorithm, the received echo signals are processed by a cross-correlation operation, thus the energy from different scatterers of a target can be accumulated. The proposed scheme can therefore work well in low SNR conditions. In the proposed algorithm, the angle estimation problem is converted to estimating the frequency of the CCF. The simulation results demonstrate the similar performance of the proposed algorithm compared with the amplitude comparison angle estimation. It means that the proposed method for angle estimation can be adopted. In modern radar systems, narrowband signals and wideband signals should be transmitted alternatively. However, when adopting the proposed method, future radars may only need wideband signals for both tracking and imaging, which can greatly increase the data rate and strengthen the capability of anti-jamming. More importantly, the estimated angle will not become ambiguous under an arbitrary angle, which can significantly extend the estimated angle range in wideband radars.
